# Incremental value of laboratory and ultrasonographic markers for preoperative risk stratification of difficult laparoscopic cholecystectomy: a cross-validated cohort study

**DOI:** 10.3389/fsurg.2026.1982578

**Published:** 2026-10-13

**Authors:** Mustafa Kağan Başdoğan, Furkan Saydın, Cem Batuhan Ofluoğlu, Güney Özkaya, Fırat Mülküt

**Affiliations:** Sancaktepe Şehit Prof. Dr. İlhan Varank Training and Research Hospital, Republic of Türkiye Ministry of Health, University of Health Sciences, Istanbul, Türkiye

**Keywords:** C-reactive protein, difficult cholecystectomy, gallbladder wall thickness, laparoscopic cholecystectomy, prediction model, preoperative risk stratification, surgical outcomes, ultrasonography

## Abstract

**Background:**

Difficult laparoscopic cholecystectomy (LC) is associated with prolonged operative times, bile duct injuries, and increased postoperative morbidity. Reliable preoperative prediction could optimize surgical planning and patient consent, but the incremental value of adding laboratory and ultrasonographic (USG) data to routinely available clinical variables has rarely been quantified.

**Materials and methods:**

We conducted a retrospective cohort study of 648 consecutive patients undergoing LC for symptomatic gallstone disease, reported in accordance with the STROCSS 2021 guidelines and relevant TRIPOD recommendations. Difficult LC was defined *a priori* as a composite outcome (operative time ≥90 min, subtotal cholecystectomy, conversion to open surgery, fundus-first dissection, or additional ports), capturing distinct but overlapping dimensions of operative difficulty ranging from prolonged dissection to surgeon-driven bailout strategies. Independent predictors were identified by multivariable logistic regression, and discriminative performance of clinical, clinical + laboratory, and combined (clinical + laboratory + USG) models was assessed using fivefold cross-validated receiver operating characteristic analysis, with bootstrap-derived 95% confidence intervals (CIs), calibration, and bootstrap optimism correction.

**Results:**

Difficult LC occurred in 125 patients (19.3%). Impacted stone [adjusted odds ratio (OR) 2.86, 95% CI 1.66–4.92], previous cholecystitis attack (OR 1.87, 95% CI 1.15–3.02), gallbladder wall thickness (OR 1.74 per mm, 95% CI 1.36–2.21), body mass index (OR 1.05 per kg/m^2^, 95% CI 1.00–1.11), and C-reactive protein (OR 1.02 per mg/L, 95% CI 1.01–1.04) were independent predictors (all *p* < 0.05). Cross-validated areas under the receiver operating characteristic curve (AUC) values increased progressively from 0.723 (95% CI 0.687–0.786) (clinical) to 0.734 (95% CI 0.700–0.796) (clinical + laboratory) to 0.768 (95% CI 0.742–0.831) (combined + USG), a pattern preserved after excluding operative time from the outcome definition [0.705 (95% CI 0.635–0.801) → 0.716 (95% CI 0.641–0.808) → 0.739 (95% CI 0.675–0.832)]. The paired-bootstrap increment in AUC was +0.011 (95% CI −0.007 to +0.040) for laboratory variables and +0.034 (95% CI +0.010 to +0.071) for ultrasonographic variables, indicating that the laboratory increment alone was not statistically distinguishable from zero, whereas the ultrasonographic increment was. The combined model showed acceptable calibration (slope 0.76, intercept 0.03, Brier score 0.134) and an optimism-corrected AUC of 0.783. Difficult LC was associated with more postoperative bile leaks, 30-day readmissions, and longer hospital stays (all *p* < 0.001).

**Conclusion:**

Sequentially adding laboratory and then ultrasonographic data to a clinical-only model produced modest, calibration-confirmed improvements in cross-validated discrimination of difficult LC. This increment was statistically robust for ultrasonographic variables (95% CI excluding zero) but not for laboratory variables alone, indicating a modest incremental improvement in discrimination rather than a clinically transformative gain. External, prospective validation is warranted before routine clinical implementation.

## 1 Introduction

Laparoscopic cholecystectomy (LC) is one of the most frequently performed procedures in general surgery and remains the standard of care for symptomatic gallstone disease and acute cholecystitis (1). Although most cases proceed uneventfully, a substantial subset—reported in up to a quarter of operations—proves technically demanding, with dense adhesions, an obscured Calot's triangle, or an inflamed, friable gallbladder wall that increases the risk of bleeding, bile duct injury (BDI), and conversion to open surgery (2). Difficult LC is consistently associated with longer operative times, greater blood loss, higher complication and conversion rates, prolonged hospitalization, and increased cost (2, 3).

Multiple preoperative scoring systems have been proposed to anticipate operative difficulty before the first incision. The Randhawa and Pujahari score—one of the earliest and most widely validated systems—combines history, clinical examination, and sonographic findings to stratify patients into easy, difficult, and very difficult categories (4). This score has since been externally validated in several populations with areas under the receiver operating characteristic curve (AUC) ranging from 0.72 to 0.86 (5, 6), most recently in a 2026 prospective cohort that confirmed its clinical utility while highlighting a persistently modest sensitivity for the difficult category (7). Intraoperative grading systems—such as the Sugrue/European Association for Endoscopic Surgery (EAES) difficulty grading scale—complement these preoperative tools by standardizing the reporting of operative findings (8). Large prospective cohorts, including a validated risk score derived from 8,820 patients in the United Kingdom, have confirmed that age, male sex, body mass index (BMI), acute cholecystitis, and sonographic gallbladder wall thickening are consistently associated with conversion and operative difficulty (9).

Systemic inflammatory markers have more recently been incorporated into predictive models. The C-reactive protein (CRP)-to-albumin ratio (CAR) has been shown to independently predict both conversion to open surgery and difficult LC in patients with acute cholecystitis diagnosed by Tokyo Guidelines 2018 (TG18) criteria (10–12). The neutrophil-to-lymphocyte ratio (NLR) has similarly been linked to cholecystitis severity (13). The Tokyo Guidelines 2018 additionally formalized the concept of “bailout” procedures—principally subtotal cholecystectomy—as an accepted strategy to avoid BDI when the critical view of safety cannot be achieved in a hostile hepatocystic triangle (14), with subsequent meta-analyses confirming that subtotal cholecystectomy reduces the risk of common bile duct (CBD) injury at the cost of a higher rate of bile leak and reoperation (15).

Despite this body of evidence, no single model combining clinical, laboratory, and ultrasonographic (USG) data has been consistently validated across settings, and most published scores have been developed in cohorts restricted to acute cholecystitis or single-center emergency populations (2, 10). The incremental value of adding routine preoperative USG findings to clinical and laboratory data—rather than considering these domains in isolation—has rarely been quantified using cross-validated discrimination metrics, together with a formal quantification of the accompanying statistical uncertainty. We therefore analyzed a cohort of 648 patients undergoing LC to (i) identify independent preoperative predictors of difficult LC, spanning clinical, laboratory, and USG domains; (ii) quantify the incremental discriminative value of sequentially adding laboratory and USG data to a clinical-only model, together with 95% confidence intervals (CIs) for each model and each pairwise increment; and (iii) characterize the postoperative morbidity attributable to difficult LC. We hypothesized that laboratory and, particularly, ultrasonographic markers reflecting systemic and local biliary inflammation would provide incremental predictive information beyond routinely available clinical variables alone.

## 2 Materials and methods

### 2.1 Study design, setting, and reporting

We conducted a retrospective cohort study of consecutive adult patients who underwent LC for symptomatic gallstone disease at the Department of General Surgery, S.B.Ü. Sancaktepe Şehit Prof. Dr. İlhan Varank Training and Research Hospital, İstanbul, Türkiye, between 01 January 2024 and 01 June 2026. The study was reported in accordance with the Strengthening the Reporting of Cohort, Cross-Sectional and Case–Control Studies in Surgery (STROCSS 2021) guidelines (16)—which build on the STROBE statement for observational studies (17) (complete checklist provided as Supplementary Table S4). Furthermore, because this work develops and internally validates a multivariable prediction model, it adheres to the relevant recommendations of the Transparent Reporting of a multivariable prediction model for Individual Prognosis Or Diagnosis (TRIPOD) statement (complete checklist provided as Supplementary Table S3). This retrospective observational cohort study was not registered in a public trial registry, consistent with local institutional policies for studies of this design. The study was approved by the Scientific Research Ethics Committee of the Sancaktepe Şehit Prof. Dr. İlhan Varank Training and Research Hospital (approval no. 2026/452, approved 26 August 2026) and conducted in accordance with the Declaration of Helsinki. The requirement for individual informed consent was waived by the Ethics Committee given the retrospective, de-identified nature of the data.

### 2.2 Participants

All patients undergoing elective or emergency LC for symptomatic cholelithiasis or acute cholecystitis during the study period were eligible. Exclusion criteria were concomitant common bile duct exploration for choledocholithiasis diagnosed preoperatively, suspected or confirmed gallbladder malignancy, planned open cholecystectomy from the outset, and missing preoperative laboratory or ultrasonographic data. A total of 648 patients met the inclusion criteria and had complete data for all analyzed variables (0 missing values).

### 2.3 Outcome definition

The primary outcome, difficult LC, was defined *a priori* as a composite endpoint present if the patient met at least one of the following intraoperative criteria: (i) total operative time ≥90 min, (ii) subtotal (partial) cholecystectomy, (iii) conversion to open surgery, (iv) fundus-first (dome-down) dissection technique, or (v) need for additional trocar placement beyond the standard four ports. This pragmatic, multi-criterion definition was chosen to capture the range of intraoperative adaptations that surgeons make in response to a hostile operative field, consistent with definitions used in prior difficulty-scoring literature (4, 18).

The five chosen components were selected because they captured distinct, partially overlapping dimensions of operative difficulty rather than a single underlying construct: Prolonged operative time and the need for additional trocars primarily reflect technical complexity and a prolonged, effortful dissection; subtotal cholecystectomy and fundus-first dissection represent surgeon-driven bailout strategies adopted when the critical view of safety cannot be achieved; and conversion to open surgery represents the most severe manifestation of intraoperative difficulty. The ≥90-min operative time threshold was not chosen arbitrarily; it corresponds to the threshold most consistently applied in the contemporary difficult LC literature, including a recent narrative review that explicitly defines difficult LC as a procedure requiring an operative time greater than 90 min, among other criteria (19). A *post hoc* sensitivity analysis excluding the operative time criterion entirely, to test the robustness of this composite definition, is reported in Section 3.6.

### 2.4 Preoperative variables

Preoperative variables were grouped into three domains for sequential modeling: (1) clinical—age, sex, BMI, American Society of Anesthesiologists (ASA) class, diabetes mellitus, hypertension, acute cholecystitis at presentation, previous cholecystitis attack, previous endoscopic retrograde cholangiopancreatography (ERCP), and previous upper abdominal surgery; (2) laboratory—white blood cell count (WBC), CRP, serum albumin, NLR, and the CRP-to-albumin ratio (CAR); and (3) ultrasonographic—gallbladder wall thickness, pericholecystic fluid, impacted stone in the neck/Hartmann's pouch, maximum stone size, and CBD diameter. Variable definitions and coding are summarized in the accompanying codebook (Supplementary Table S1).

### 2.5 Statistical analysis

Continuous variables are presented as mean ± standard deviation and compared between difficult and non-difficult groups using Student's two-sample t-test with Welch's correction for unequal variances. Categorical variables are presented as *n* (%) and compared using the chi-square test, or Fisher's exact test when any expected cell count was <5. All tests were two-sided, with statistical significance set at *p* < 0.05; no adjustment for multiple comparisons was applied to the exploratory univariable screen, consistent with its role in variable selection rather than confirmatory inference.

Variables that were significant on univariable analysis or judged clinically important *a priori* were entered into a multivariable logistic regression model with difficult LC as the dependent variable.

To make this selection process fully transparent, seven of the nine variables entered into the multivariable model reached conventional univariable significance (*p* < 0.05 in Table 1): acute cholecystitis at admission, previous cholecystitis attack, impacted stone, pericholecystic fluid, gallbladder wall thickness, NLR, and CRP. Two additional variables—BMI (univariable *p* = 0.063) and previous ERCP (univariable *p* = 0.083)—did not reach conventional significance in this cohort but were retained *a priori* because of their established and reproducible role as predictors of operative difficulty in prior, larger cohorts (9–11). No variables were added *post hoc* on the basis of the multivariable results; the *a priori* and univariable-driven components of the selection process were fixed before model fitting.

**Table 1:** Baseline clinical, laboratory, and ultrasonographic characteristics by surgical difficulty group.

Variable	Non-difficult (*n* = 523)	Difficult (*n* = 125)	*p*-value
Age (years)	52.2 ± 14.5	51.3 ± 14.9	0.565
Sex, male	213 (40.7%)	54 (43.2%)	0.686
BMI (kg/m^2^)	28.0 ± 4.5	28.8 ± 4.3	0.063
Diabetes mellitus	214 (40.9%)	45 (36.0%)	0.365
Hypertension	136 (26.0%)	31 (24.8%)	0.871
Acute cholecystitis at admission	180 (34.4%)	82 (65.6%)	<0.001
Previous cholecystitis attack	150 (28.7%)	74 (59.2%)	<0.001
Previous ERCP	63 (12.0%)	23 (18.4%)	0.083
Previous upper abdominal surgery	43 (8.2%)	14 (11.2%)	0.379
WBC (10^9^/L)	8.6 ± 2.7	9.6 ± 2.8	<0.001
CRP (mg/L)	11.3 ± 12.7	24.1 ± 25.5	<0.001
Albumin (g/dL)	4.1 ± 0.4	4.1 ± 0.3	0.605
NLR	3.1 ± 1.7	4.1 ± 2.3	<0.001
CAR	2.9 ± 3.4	6.0 ± 6.6	<0.001
Gallbladder wall thickness (mm)	3.3 ± 1.3	4.5 ± 1.1	<0.001
Pericholecystic fluid	45 (8.6%)	19 (15.2%)	0.040
Impacted stone	56 (10.7%)	38 (30.4%)	<0.001
Max stone size (mm)	13.7 ± 5.8	14.5 ± 5.7	0.163
CBD diameter (mm)	5.4 ± 1.5	5.7 ± 1.5	0.098

Data are presented as mean ± standard deviation for continuous variables (Student's t-test) or *n* (%) for categorical variables (chi-square or Fisher's exact test).

Results are reported as adjusted odds ratios (ORs) with 95% CIs and Wald *p*-values. Model discrimination was assessed by McFadden's pseudo-R^2^ and by the concordance (c-statistic/AUC) of the fitted model.

To quantify the incremental value of each variable domain, three nested logistic regression models were constructed: a clinical model, a clinical + laboratory model, and a combined (clinical + laboratory + USG) model. To avoid optimistic bias from in-sample evaluation, each model was evaluated using stratified fivefold cross-validation: In each fold, predictors were standardized and the model was fitted on 80% of the data and applied to the held-out 20%, with out-of-fold predicted probabilities pooled across all five folds to compute the receiver operating characteristic curve and AUC. The Youden index was used to select the optimal probability threshold, from which sensitivity and specificity were derived.

Uncertainty around each cross-validated AUC was quantified with a patient-level bootstrap procedure (1,000 resamples with replacement). For each resample, the entire stratified fivefold cross-validation pipeline—predictor standardization, model fitting, out-of-fold prediction, and AUC computation—was repeated on the resampled cohort, and the 2.5th and 97.5th percentiles of the resulting distribution of AUC values were taken as the 95% CI. Uncertainty around the incremental AUC gain attributable to each variable domain (ΔAUC) was estimated using a paired bootstrap: For each of the 1,000 resamples, all three nested models were evaluated on the same resampled dataset, so that the pairwise AUC differences (clinical → clinical + laboratory, clinical + laboratory → combined, and clinical → combined) were computed on identical data in every resample; the 95% CI for each ΔAUC was then derived from the 2.5th and 97.5th percentiles of the corresponding bootstrap distribution of differences. This paired approach preserves the correlation between nested models evaluated on the same patients and yields a valid, generally narrower confidence interval for the pairwise comparison than would be obtained from independent bootstrap samples for each model.

Secondary intraoperative and postoperative outcomes [operative time, subtotal cholecystectomy, conversion, drain placement, gallbladder perforation, bile leak, intra-abdominal collection, postoperative ERCP, reoperation, length of stay, 30-day readmission, major complication (Clavien–Dindo grade ≥ III) (20), and 30-day mortality] were compared between groups using the same univariable methods described earlier.

All analyses were performed in Python 3.11 using pandas, SciPy, statsmodels, and scikit-learn. Figures were generated using Matplotlib. A two-sided *p*-value <0.05 was considered statistically significant throughout.

### 2.6 Sensitivity analysis, calibration, subgroup analysis, and exploratory risk score development

Because operative time ≥90 min both contributed to the composite difficult LC definition and was subsequently reported as a group comparison outcome, we performed a *post hoc* sensitivity analysis in which difficult LC was redefined using only the four non-temporal criteria (subtotal cholecystectomy, conversion to open surgery, fundus-first dissection, or additional port placement), excluding operative time entirely. The three nested models and multivariable logistic regression were refitted under this alternative definition using the same fivefold cross-validation procedure, and 95% bootstrap confidence intervals were computed for each of the three resulting AUCs using the identical procedure described in Section 2.5.

Calibration of the combined model was assessed using pooled out-of-fold predicted probabilities from the fivefold cross-validation. Calibration slope and calibration-in-the-large (intercept) were estimated by logistic recalibration of observed outcomes on the logit of predicted probability, and overall calibration accuracy was summarized with the Brier score. To further characterize optimism in the apparent (in-sample) discrimination, we performed bootstrap optimism correction with 300 bootstrap resamples: The model was refitted in each bootstrap sample, evaluated on both the bootstrap sample and the original cohort, and the average difference in AUC (optimism) was subtracted from the apparent AUC of the full-data model.

As an exploratory *post hoc* analysis requested during peer review, we additionally evaluated the combined model separately in patients with and without acute cholecystitis at presentation, using the same cross-validated bootstrap procedure described in Section 2.5. Because acute cholecystitis status is, by construction, invariant within each subgroup, this predictor was excluded from the subgroup-specific models to avoid a zero-variance covariate; all other clinical, laboratory, and ultrasonographic predictors were retained. Given the reduced sample size and event count within each subgroup relative to the full cohort, this analysis is explicitly exploratory and hypothesis-generating rather than confirmatory. Information on elective-vs.-emergency operative setting was not reliably or consistently recorded in the underlying institutional records for this retrospective cohort and could therefore not be analyzed; this is discussed as a limitation in Section 4.1.

To illustrate potential clinical usability, we constructed an exploratory integer point-based risk score from the five independent predictors of the primary multivariable model (impacted stone, gallbladder wall thickness, previous cholecystitis attack, CRP, and BMI), with points assigned proportionally to each variable's regression coefficient, anchored to previous cholecystitis attack (two points). This score is presented as a preliminary illustration of a simplified clinical tool and requires prospective calibration and validation before any clinical use.

## 3 Results

### 3.1 Study population

A total of 648 patients undergoing LC were analyzed, with no missing data for any of the 36 preoperative, intraoperative, or postoperative variables recorded. Difficult LC, as defined by the composite primary outcome, occurred in 125 patients (19.3%); the remaining 523 patients (80.7%) underwent a non-difficult (standard) procedure.

### 3.2 Preoperative predictors of difficult LC (univariable analysis)

Table 1 summarizes preoperative clinical, laboratory, and ultrasonographic characteristics by group. Patients who developed difficult LC were not older or more frequently male than those with a non-difficult course. Occurrence of diabetes and hypertension, BMI, and albumin did not differ significantly between groups. In contrast, acute cholecystitis at admission (65.6% vs. 34.4%), a previous cholecystitis attack (59.2% vs. 28.7%), and an impacted stone on USG (30.4% vs. 10.7%) were markedly more common in the difficult LC group (all *p* < 0.001). Inflammatory markers were also significantly elevated in the difficult LC group, including WBC (9.6 ± 2.8 × 10^9^/L vs. 8.6 ± 2.7 × 10^9^/L), CRP (24.1 ± 25.5 mg/L vs. 11.3 ± 12.7 mg/L), NLR (4.1 ± 2.3 vs. 3.1 ± 1.7), and the CRP/albumin ratio (6.0 ± 6.6 vs. 2.9 ± 3.4; all *p* < 0.001). Sonographically, gallbladder wall thickness was greater in the difficult LC group (4.5 ± 1.1 mm vs. 3.3 ± 1.3 mm, *p* < 0.001), as was the presence of pericholecystic fluid (15.2% vs. 8.6%, *p* = 0.040).

### 3.3 Independent predictors: multivariable logistic regression

On multivariable logistic regression (Table 2, Figure 1), five variables remained independently associated with difficult LC after adjustment: impacted stone (adjusted OR 2.86, 95% CI 1.66–4.92; *p* < 0.001), gallbladder wall thickness (adjusted OR 1.74 per mm, 95% CI 1.36–2.21; *p* < 0.001), previous cholecystitis attack (adjusted OR 1.87, 95% CI 1.15–3.02; *p* = 0.011), CRP (adjusted OR 1.02 per mg/L, 95% CI 1.01–1.04; *p* = 0.003), and BMI (adjusted OR 1.05 per kg/m^2^, 95% CI 1.00–1.11; *p* = 0.034). Acute cholecystitis at admission, NLR, pericholecystic fluid, and previous ERCP were not independently associated with difficult LC once these five variables were accounted for. The model demonstrated acceptable fit (McFadden's pseudo-R^2^ = 0.190; likelihood ratio *p* < 0.001), indicating that a substantial portion of the variance in surgical difficulty was explained by preoperative variables, while unmeasured intraoperative factors presumably account for the remainder.

**Table 2:** Independent predictors of difficult laparoscopic cholecystectomy: multivariable logistic regression.

Variable	Adjusted OR (95% CI)	*p*-value
Impacted stone (yes vs. no)	2.86 (1.66–4.92)	<0.001
Previous cholecystitis attack (yes vs. no)	1.87 (1.15–3.02)	0.011
Gallbladder wall thickness (per 1 mm)	1.74 (1.36–2.21)	<0.001
Previous ERCP (yes vs. no)	1.20 (0.65–2.20)	0.557
NLR (per 1 unit)	1.10 (0.97–1.25)	0.147
Pericholecystic fluid (yes vs. no)	1.09 (0.56–2.12)	0.796
BMI (per 1 kg/m^2^)	1.05 (1.00–1.11)	0.034
CRP (per 1 mg/L)	1.02 (1.01–1.04)	0.003
Acute cholecystitis at admission (yes vs. no)	0.59 (0.29–1.19)	0.138

**Figure 1:**
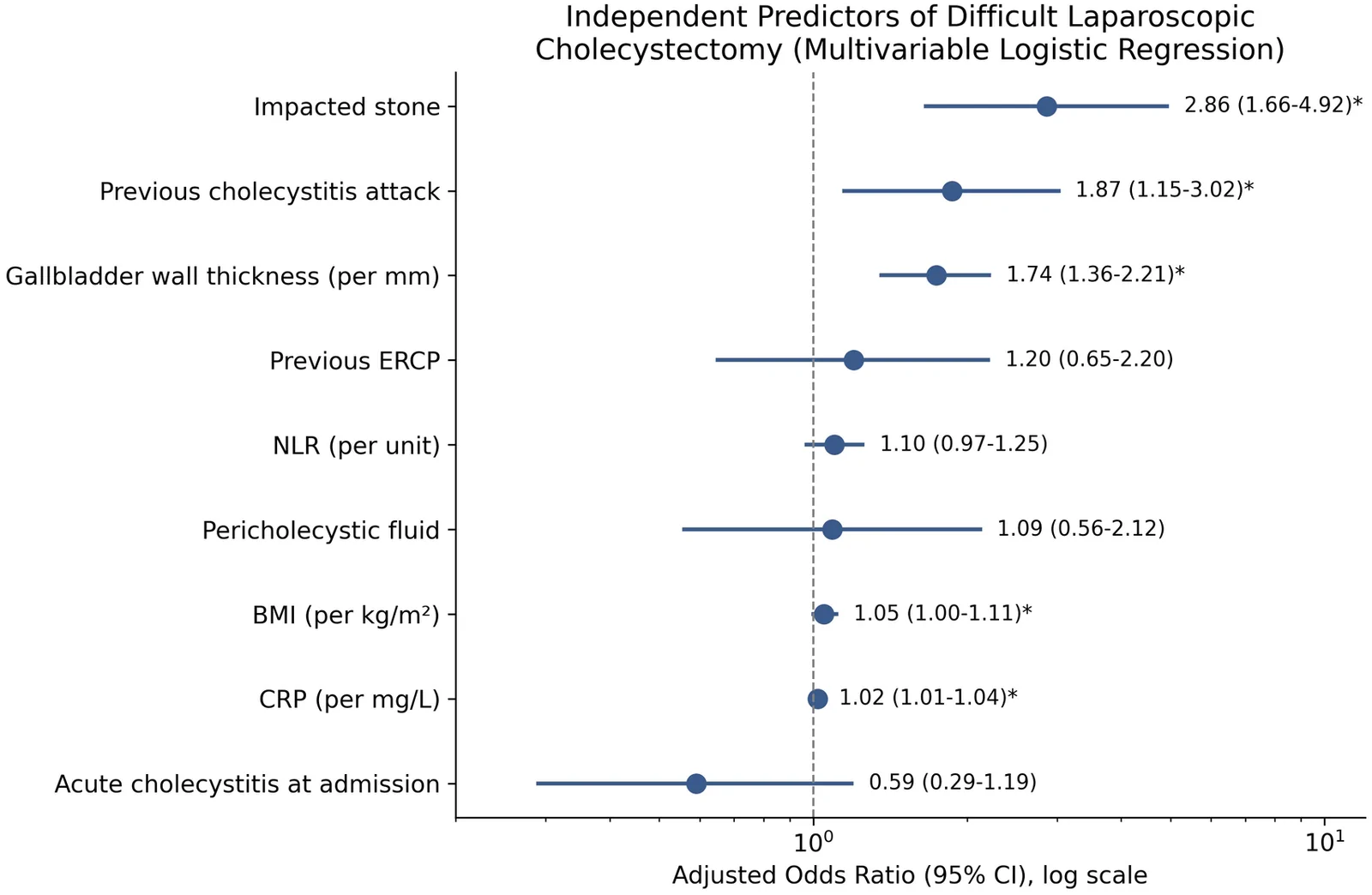
Forest plot of adjusted odds ratios (95% CI) for independent predictors of difficult laparoscopic cholecystectomy. Asterisks denote *p* < 0.05.

### 3.4 Incremental discriminative value of clinical, laboratory, and ultrasonographic data

Discriminative performance of the three nested, fivefold cross-validated models is shown in Table 3 and Figure 2. The clinical-only model achieved an AUC of 0.723 (95% CI 0.687–0.786) (sensitivity 77.6%, specificity 61.6% at the Youden-optimal threshold). Addition of laboratory variables (WBC, CRP, albumin, NLR) modestly improved discrimination to an AUC of 0.734 (95% CI 0.700–0.796). The combined model, incorporating ultrasonographic variables (gallbladder wall thickness, pericholecystic fluid, impacted stone, maximum stone size, and CBD diameter) in addition to clinical and laboratory data, achieved the highest discrimination [AUC 0.768 (95% CI 0.742–0.831); sensitivity 76.0%, specificity 66.5%].

**Table 3:** Discriminative performance of nested prediction models (fivefold cross-validated), with bootstrap 95% confidence intervals.

Model	Cross-validated AUC (95% CI)	ΔAUC (95% CI)	Sensitivity (%)	Specificity (%)
Clinical	0.723 (0.687–0.786)	Reference	77.6	61.6
Clinical + Laboratory	0.734 (0.700–0.796)	+0.011 (−0.007 to +0.040)	78.4	59.8
Combined + USG	0.768 (0.742–0.831)	+0.034 (+0.010 to +0.071) vs. Clin + Lab +0.045 (+0.020 to +0.091) vs. clinical	76.0	66.5

AUC and ΔAUC 95% CIs derived from 1,000 patient-level bootstrap resamples of the full stratified fivefold cross-validation pipeline (Section 2.5). Sensitivity and specificity reported at the Youden-optimal probability threshold, derived from pooled out-of-fold predictions across five cross-validation folds.

**Figure 2:**
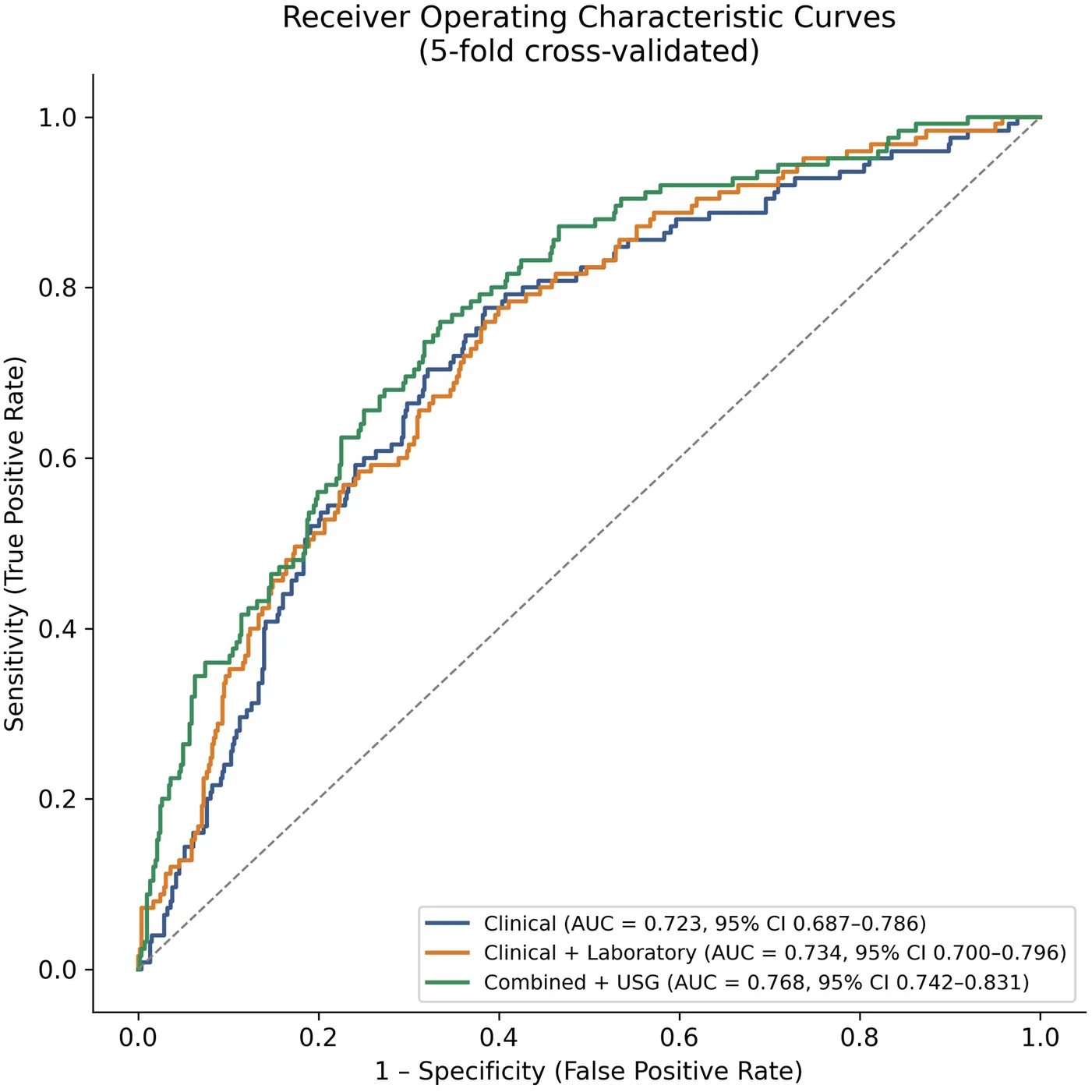
Fivefold cross-validated ROC curves for the clinical, clinical + laboratory, and combined (+USG) models (AUCs 0.723, 0.734, and 0.768, respectively).

Using a paired bootstrap procedure that evaluated all three nested models on the same 1,000 resampled datasets (Section 2.5), the corresponding ΔAUC was +0.011 (95% CI −0.007 to +0.040) for the addition of laboratory variables to the clinical model, +0.034 (95% CI +0.010 to +0.071) for the addition of ultrasonographic variables to the clinical + laboratory model, and +0.045 (95% CI +0.020 to +0.091) for the combined model vs. the clinical-only baseline (Table 3). The 95% CI for the laboratory-only increment included zero, indicating that this small numerical gain was not statistically distinguishable from no improvement, whereas the increments attributable to ultrasonographic data and to the combined model were both statistically supported (95% CI excluding zero).

*Post hoc* sensitivity analysis and calibration assessment for the combined model are reported in Sections 3.6 and 3.7; exploratory subgroup analysis by acute cholecystitis status is reported in Section 3.8; and an exploratory, non-validated preliminary risk score derived from the model is presented in Supplementary Table S2.

### 3.5 Intraoperative and postoperative outcomes

Table 4 summarizes intraoperative and postoperative outcomes. As expected from the outcome definition, operative time was markedly longer in the difficult LC group (106.9 ± 20.4 min vs. 53.6 ± 13.3 min, *p* < 0.001); subtotal cholecystectomy (13.6% vs. 0%), conversion to open surgery (4.8% vs. 0%), fundus-first dissection (17.6% vs. 0%), and additional port placement (13.6% vs. 0%) occurred exclusively or almost exclusively within this group by definition. Beyond these definitional criteria, difficult LC was associated with substantially higher rates of intraoperative gallbladder perforation (28.8% vs. 9.6%, *p* < 0.001) and drain placement (60.8% vs. 16.1%, *p* < 0.001). Postoperatively, the difficult LC group experienced more bile leaks (4.8% vs. 0.4%, *p* < 0.001), more postoperative ERCP (4.8% vs. 0.6%, *p* = 0.002), more reoperations (4.0% vs. 0%, *p* < 0.001), longer hospital stays (3.8 ± 1.5 vs. 2.0 ± 1.0 days, *p* < 0.001), higher 30-day readmissions (8.8% vs. 1.7%, *p* < 0.001), and more major complications graded Clavien–Dindo ≥ III (8.8% vs. 0.8%, *p* < 0.001). Thirty-day mortality was rare in both groups (0.8% vs. 0%, *p* = 0.193) and did not reach statistical significance in this cohort.

**Table 4:** Intraoperative and postoperative outcomes by surgical difficulty group.

Variable	Non-difficult (*n* = 523)	Difficult (*n* = 125)	*p*-value
Operative time (min)	53.6 ± 13.3	106.9 ± 20.4	<0.001
Subtotal cholecystectomy	0 (0.0%)	17 (13.6%)	<0.001
Conversion to open surgery	0 (0.0%)	6 (4.8%)	<0.001
Fundus-first technique	0 (0.0%)	22 (17.6%)	<0.001
Additional port use	0 (0.0%)	17 (13.6%)	<0.001
Drain placement	84 (16.1%)	76 (60.8%)	<0.001
Gallbladder perforation	50 (9.6%)	36 (28.8%)	<0.001
Postoperative bile leak	2 (0.4%)	6 (4.8%)	<0.001
Intra-abdominal collection	2 (0.4%)	3 (2.4%)	0.052
Postoperative ERCP	3 (0.6%)	6 (4.8%)	0.002
Reoperation	0 (0.0%)	5 (4.0%)	<0.001
Length of stay (days)	2.0 ± 1.0	3.8 ± 1.5	<0.001
30-day readmission	9 (1.7%)	11 (8.8%)	<0.001
Major complication (Clavien–Dindo ≥ III)	4 (0.8%)	11 (8.8%)	<0.001
30-day mortality	0 (0.0%)	1 (0.8%)	0.193

### 3.6 Sensitivity analysis excluding operative time from the outcome definition

Because operative time contributed to both the outcome definition and a subsequently reported group comparison, we repeated the primary analysis using a stricter, non-temporal definition of difficult LC (subtotal cholecystectomy, conversion, fundus-first dissection, or additional port placement only). Under this definition, 52 patients (8.0%) met criteria for difficult LC. The incremental pattern of discrimination was preserved: cross-validated AUC increased from 0.705 (95% CI 0.635–0.801) (clinical) to 0.716 (95% CI 0.641–0.808) (clinical + laboratory) to 0.739 (95% CI 0.675–0.832) (combined + USG), with ultrasonographic variables again contributing the largest incremental gain (Table 5). On multivariable analysis of this alternative outcome, gallbladder wall thickness (adjusted OR 1.83, 95% CI 1.30–2.57; *p* < 0.001), impacted stone (adjusted OR 2.08, 95% CI 1.04–4.16; *p* = 0.037), and BMI (adjusted OR 1.09, 95% CI 1.02–1.17; *p* = 0.010) remained independent predictors, whereas previous cholecystitis attack and CRP were no longer statistically significant, likely reflecting their stronger association with prolonged operative time specifically. This confirms that the incremental value of sequentially adding laboratory and USG data is not an artifact of including operative time in the primary outcome definition, although the specific contribution of inflammatory markers (CRP) appears more closely tied to the operative time component of difficulty.

**Table 5:** Sensitivity analysis: cross-validated model discrimination using a non-temporal definition of difficult LC, with bootstrap 95% confidence intervals.

Model	AUC (95% CI)	Sensitivity (%)	Specificity (%)
Clinical	0.705 (0.635–0.801)	63.5	76.2
Clinical + Laboratory	0.716 (0.641–0.808)	76.9	65.3
Combined (+USG)	0.739 (0.675–0.832)	80.8	59.6

Difficult LC redefined as subtotal cholecystectomy, conversion to open surgery, fundus-first dissection, or additional port placement (*n* = 52, 8.0%), excluding the operative time criterion. AUC 95% CIs derived from 1,000 patient-level bootstrap resamples of the full cross-validation pipeline. Sensitivity and specificity reported at the Youden-optimal threshold, fivefold cross-validated, pooled out-of-fold predictions.

### 3.7 Calibration and optimism-corrected discrimination of the combined model

Calibration of the combined model, assessed on pooled out-of-fold predicted probabilities, showed a calibration intercept (calibration-in-the-large) of 0.03 (*p* = 0.83), indicating no significant systematic over- or under-prediction of risk across the cohort. The calibration slope was 0.76 (*p* < 0.001 vs. the ideal value of 1), indicating mild overfitting, with predicted probabilities modestly too extreme at the high and low ends of the risk range (Figure 3). Overall calibration accuracy was acceptable (Brier score 0.134, where 0 indicates perfect prediction). Bootstrap optimism correction (300 resamples) yielded an apparent (in-sample) AUC of 0.816 and a mean optimism of 0.033, giving an optimism-corrected AUC of 0.783—closely consistent with the fivefold cross-validated estimate of 0.768 and supporting the robustness of the cross-validation approach used as the primary discrimination metric.

**Figure 3:**
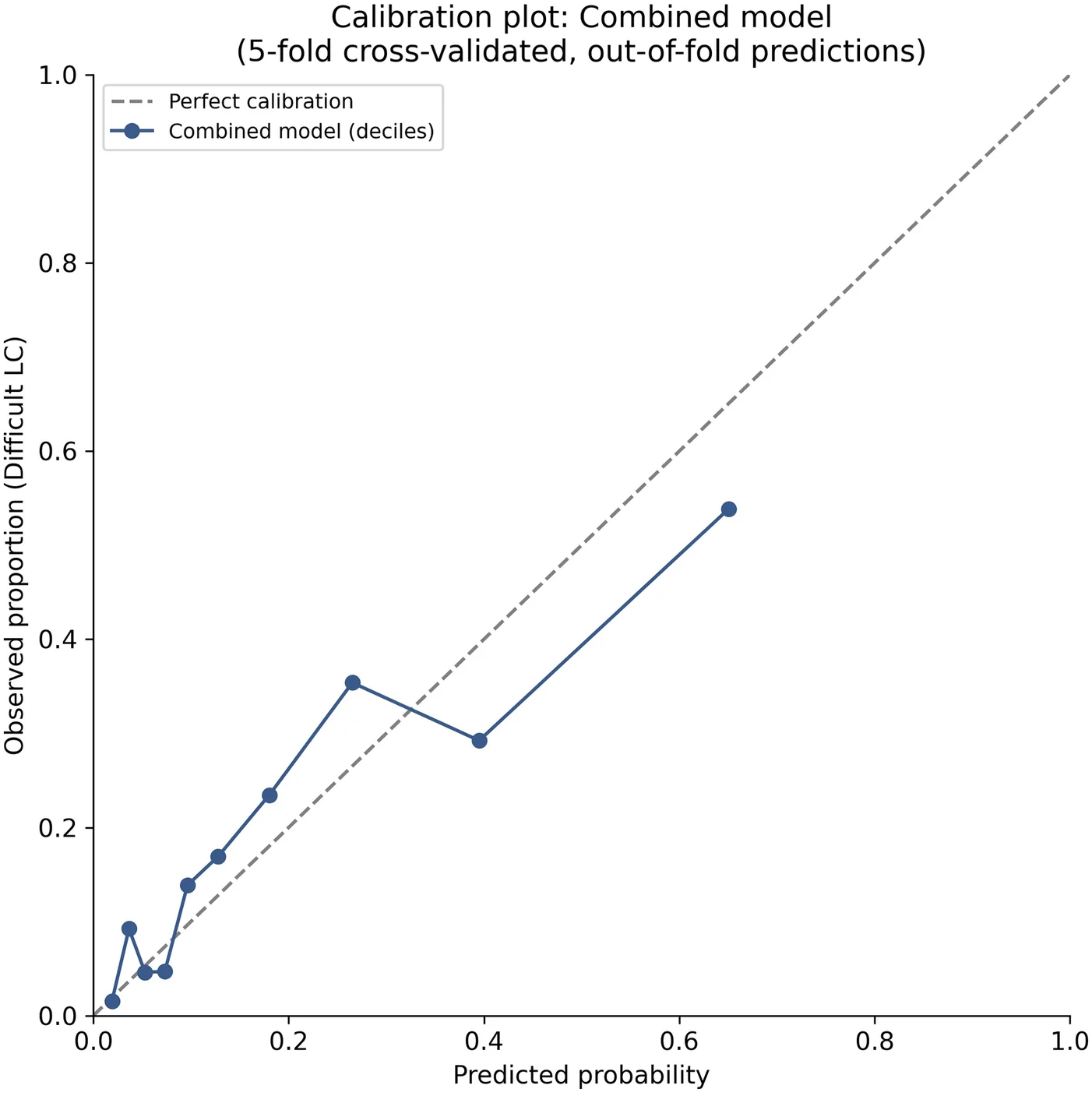
Calibration plot for the combined (clinical + laboratory + USG) model, based on pooled out-of-fold predictions from fivefold cross-validation, grouped into deciles of predicted risk.

An exploratory, illustrative point-based risk score derived from the five independent predictors of the primary multivariable model, together with its apparent discrimination and quartile-stratified event rates, is presented in Supplementary Material Table S2; this score was not externally validated and is not intended to guide clinical decisions in its current form.

### 3.8 Exploratory subgroup analysis by acute cholecystitis status

Reviewer 2 raised the possibility that model performance might be driven disproportionately by a particular operative-setting subgroup, given that the cohort included both acute and non-acute presentations. To address this, we performed an exploratory *post hoc* subgroup analysis of the combined model according to acute cholecystitis status at presentation, using the same cross-validated bootstrap procedure described in Section 2.5 (the Acute_Cholecystitis predictor itself was excluded from each subgroup-specific model, as it is invariant within each subgroup). Among the 262 patients with acute cholecystitis at admission (82 difficult LC, 31.3%), the combined model achieved a cross-validated AUC of 0.706 (95% CI 0.685–0.832). Among the 386 patients without acute cholecystitis at admission (43 difficult LC, 11.1%), the AUC was 0.666 (95% CI 0.641–0.820).

Both subgroup point estimates were numerically lower than, but broadly overlapping with, the AUC obtained in the full cohort (0.768), suggesting that discrimination was not confined to, or driven disproportionately by, a single high-risk operative subgroup. However, the wide and overlapping confidence intervals—driven by the smaller subgroup sample sizes and event counts relative to the full cohort—preclude any firm statistical conclusion about a true difference in performance between the two settings, and this analysis should be interpreted as exploratory and hypothesis-generating rather than confirmatory. Information on elective-vs.-emergency operative setting was not reliably or consistently recorded in the underlying institutional records for this retrospective cohort and could therefore not be analyzed as an additional stratification variable; this represents an additional, unaddressed source of potential heterogeneity that is discussed as a limitation in Section 4.1. Full bootstrap distributions for this subgroup analysis are provided in Supplementary Table S6. The results of the exploratory subgroup analysis are summarized in Table 6.

**Table 6:** Exploratory subgroup analysis of the combined model according to acute cholecystitis status at presentation.

**Subgroup**	**n**	**Difficult LC, n (%)**	**Cross-validated AUC (95% CI)**
Acute cholecystitis present	262	82 (31.3%)	0.706 (0.685–0.832)
Acute cholecystitis absent	386	43 (11.1%)	0.666 (0.641–0.820)
Full cohort (for reference)	648	125 (19.3%)	0.768 (0.742–0.831)

Acute_Cholecystitis excluded from subgroup-specific models (invariant within subgroup). Exploratory analysis; interpret cautiously given reduced subgroup sample sizes. Elective/emergency operative setting was not available in this dataset and could not be analyzed (see Limitations).

## 4 Discussion

In this cohort of 648 patients, difficult LC occurred in 19.3% of cases, consistent with prior reports of 13%–26% depending on case mix and the definition applied (2, 18). Five preoperative variables—impacted stone, gallbladder wall thickness, previous cholecystitis attack, CRP, and BMI—independently predicted difficulty, and a model combining clinical, laboratory, and ultrasonographic data outperformed a clinical-only model (cross-validated AUC 0.768 vs. 0.723). These findings support the incorporation of routine, low-cost, and widely available preoperative data into structured risk assessment for LC.

The identification of impacted stone and gallbladder wall thickness as the strongest independent predictors reproduces one of the most consistent findings in this literature. The original Randhawa and Pujahari score identified gallbladder wall thickness and impacted stone as significant contributors to a validated 15-point preoperative scale (4). Subsequent external validations in South Asian and Southeast Asian cohorts have confirmed thickened gallbladder wall, impacted stone, and a history of hospitalization for cholecystitis as recurring, reproducible predictors across populations and health systems (5, 6, 18). Our adjusted odds ratio for gallbladder wall thickness (1.74 per mm) is closely comparable to previously reported estimates in the range of 1.7–2.0 per mm, reinforcing the biological plausibility that wall thickening reflects transmural inflammation and fibrosis, which obscure the hepatocystic triangle and increases the risk of an unsafe dissection plane.

The independent contribution of CRP is consistent with a growing body of evidence linking systemic inflammatory markers to operative difficulty. Elevated CRP and a raised CRP-to-albumin ratio have each been shown to predict both difficult LC and conversion to open surgery in patients with Tokyo Guidelines-defined acute cholecystitis (10–12), and the neutrophil-to-lymphocyte ratio has similarly been correlated with cholecystitis severity in other cohorts (13). In our multivariable model, CRP retained independent significance whereas NLR and acute cholecystitis did not, suggesting that CRP may capture inflammatory burden more efficiently than a binary diagnosis of acute cholecystitis alone once other covariates are accounted for—an observation that merits confirmation in prospective, TG18-stratified cohorts.

The stepwise improvement in discrimination as laboratory and subsequent ultrasonographic variables were added to the clinical model (AUC 0.723 → 0.734 → 0.768) echoes large prospective series in which combined clinical, biochemical, and radiological scores outperformed any single domain in isolation (9, 18). However, when this incremental pattern was quantified with paired-bootstrap 95% confidence intervals (Section 3.4), the increment attributable to laboratory variables alone (ΔAUC +0.011, 95% CI −0.007 to +0.040) was small and not statistically distinguishable from zero, whereas the larger increment observed after incorporation of ultrasonographic variables (ΔAUC +0.034, 95% CI +0.010 to +0.071 vs. the clinical + laboratory model) was statistically supported. This pattern indicates that, in the present cohort, laboratory variables provided only a small numerical incremental gain, whereas the larger and more statistically robust improvement in discrimination was observed after incorporation of readily available ultrasonographic variables.

The absolute magnitude of improvement observed here—approximately 0.01 AUC units from laboratory data alone and a further 0.03 from ultrasonographic data, for a cumulative gain of 0.045 over the clinical-only model—represents a modest incremental improvement in discrimination rather than a clinically transformative gain, and this more cautious framing is preferred here in place of any characterization of the improvement as “clinically significant.” Given that USG is already performed routinely as part of the diagnostic work-up for gallstone disease and adds negligible incremental cost or delay, even a modest, statistically supported improvement in discrimination may have practical value for surgical planning at the population level; however, translating this into a genuine clinical benefit—rather than a purely statistical one—requires confirmation in prospective, externally validated cohorts before any implementation claim can be made.

Importantly, this incremental pattern persisted after removal of operative time from the composite outcome definition (Section 3.6), reducing concern that model performance was driven by the temporal component of the difficulty definition rather than by genuine anatomical and inflammatory determinants of surgical difficulty.

The postoperative consequences of difficult LC observed in our cohort—higher rates of bile leak, postoperative ERCP, reoperation, 30-day readmission, and major complications—mirror the broader literature on the downstream costs of unanticipated operative difficulty (2, 3). Notably, subtotal cholecystectomy, performed in 13.6% of the difficult LC group, was not associated with any bile duct injury in this cohort, consistent with its established role as a bailout strategy that trades a higher risk of bile leak for a markedly lower risk of the far more morbid complication of bile duct injury (14, 15). This trade-off underscores the potential clinical value of preoperative risk prediction: Patients identified as high-risk for difficulty could be preferentially scheduled with adequate operating time, assigned to surgeons experienced in bailout techniques, or counseled preoperatively about the possibility of subtotal cholecystectomy or conversion.

Clinically, these findings suggest a pragmatic, two-tier approach: routine preoperative clinical and laboratory data (age, BMI, history of cholecystitis, CRP) can flag at-risk patients at the point of triage, while targeted attention to sonographic gallbladder wall thickness and stone impaction at the time of diagnostic ultrasound can refine this estimate without requiring additional imaging. Such a model could support operating room scheduling, allocation of cases to surgeons with greater laparoscopic experience, and more informed preoperative counseling regarding the possibility of subtotal cholecystectomy or conversion.

### 4.1 Strengths and limitations

This study has several strengths, including a relatively large sample size for a single-cohort analysis of difficult LC, a pragmatic multi-criterion outcome definition capturing several recognized intraoperative markers of difficulty, and a cross-validated (rather than purely in-sample) evaluation of model discrimination that is now accompanied by bootstrap-derived 95% confidence intervals for both absolute and incremental (ΔAUC) discrimination, reducing the risk of over-interpreting small numerical differences between nested models. Nonetheless, several limitations warrant consideration. First, the retrospective, single-center design limits generalizability, and residual confounding by unmeasured factors—particularly surgeon experience, which strongly influences both the threshold for conversion and the definition of “difficult” itself—cannot be excluded (2).

Surgeon experience was not reliably or systematically recorded in the institutional records underlying this retrospective cohort and could therefore not be entered as a covariate or examined in a stratified analysis. Because more experienced surgeons may both complete technically demanding cases more efficiently and be preferentially allocated to higher-risk patients, this remains an important and unresolved source of potential residual confounding that should be prospectively and systematically captured (e.g., surgeon seniority, case volume) in future validation studies. Relatedly, information on the elective-vs.-emergency operative setting was not reliably available in this dataset and could not be analyzed; combined with the inclusion of both acute and non-acute presentations, this raises the possibility of clinically meaningful heterogeneity in the underlying difficulty construct across operative contexts. An exploratory subgroup analysis stratified by acute cholecystitis status (Section 3.8) suggested that model discrimination was not confined to a single high-risk subgroup, but the wide, overlapping confidence intervals around these subgroup estimates—driven by limited subgroup sample sizes—preclude firm conclusions, and this heterogeneity should be explicitly and prospectively addressed in future external validation cohorts with complete surgeon-experience and operative-setting data.

Second, although the composite outcome definition was specified *a priori* and grounded in prior literature, and a sensitivity analysis excluding the operative time criterion (Section 3.6) confirmed that the overall pattern of incremental discrimination was not an artifact of this definitional choice, alternative definitions (e.g., intraoperative surgeon-rated difficulty scales) may still yield different predictor sets and should be explored in future work (8). Third, internal cross-validation, while more rigorous than in-sample assessment, does not substitute for external validation in an independent cohort or health system; the reported AUCs, corroborated by bootstrap optimism correction and an acceptable calibration slope and Brier score (Section 3.7), should therefore be considered an internally validated estimate of discrimination rather than a generalizable performance guarantee. Fourth, we did not capture time from symptom onset to surgery, a factor previously associated with conversion risk (10), nor did we assess interobserver variability in sonographic measurements. Finally, the low event rates for some postoperative outcomes (e.g., mortality, intra-abdominal collection) limited statistical power for those specific comparisons.

### 4.2 Future directions

Prospective, multicenter validation of the five-variable model identified here—ideally with prespecified intraoperative difficulty grading and surgeon-experience adjustment—would strengthen the evidence base for routine clinical use. Incorporation of additional imaging biomarkers (e.g., computed tomography attenuation of the gallbladder wall) and machine learning approaches capable of modeling non-linear interactions may further improve discrimination beyond the logistic regression framework used here, as suggested by recent work applying ensemble methods to related prediction problems in biliary surgery (21, 22).

## 5 Conclusion

In this cohort of 648 patients, difficult laparoscopic cholecystectomy occurred in nearly one in five patients and was independently predicted by impacted stone, gallbladder wall thickness, previous cholecystitis attack, CRP, and BMI. Sequential addition of laboratory and ultrasonographic variables to the clinical-only model resulted in a modest improvement in cross-validated discrimination. The improvement was primarily attributable to ultrasonographic variables, whereas the incremental contribution of laboratory variables alone was not statistically significant. Difficult LC was associated with substantial postoperative morbidity. These findings support the development and prospective external validation of a simple, multi-domain preoperative risk model to guide surgical planning, case allocation, and patient counseling. Future studies should account for surgeon experience and operative-setting heterogeneity as potential sources of residual confounding.

## Guarantor

Furkan Saydın, the corresponding author, accepts full responsibility for the work and the conduct of the study, had full access to the data, and maintained complete control over the decision to publish.

## Data Availability

The original contributions presented in the study are included in the article/Supplementary Material; further inquiries can be directed to the corresponding authors.
