# Supplementary material for: Incremental value of laboratory and ultrasonographic markers for preoperative risk stratification of difficult laparoscopic cholecystectomy: a cross-validated cohort study
Source: Front Surg. 2026 Oct 13;13:1982578. doi: 10.3389/fsurg.2026.1982578 (PMC13710260; doi:10.3389/fsurg.2026.1982578)
Supplement: Supplementary file 1 [file Supplementaryfile1.docx]

**Supplementary Material**

*Incremental Value of Laboratory and Ultrasonographic Markers for Preoperative Risk Stratification of Difficult Laparoscopic Cholecystectomy: A Cross-Validated Cohort Study*

Manuscript ID 1982578 — Frontiers in Surgery, Visceral Surgery

*Contains Supplementary Tables S1–S4 (original submission) and Supplementary Tables S5–S6 (added in response to Reviewer 1 and Reviewer 2, Round 1 revision).*

# **Supplementary Table S1. Variable definitions and coding**

Definitions and coding for all variables used in the analysis, corresponding to the dataset codebook.

| **Variable** | **Definition / coding** |
| --- | --- |
| Patient_ID | Unique de-identified patient identifier |
| Age_years | Age, years |
| Sex_Male | 0 = Female, 1 = Male |
| BMI_kg_m2 | Body mass index, kg/m² |
| ASA | American Society of Anesthesiologists physical status class, 1–4 |
| Diabetes / Hypertension | 0 = No, 1 = Yes |
| Acute_Cholecystitis | Acute cholecystitis at presentation: 0/1 |
| Previous_Cholecystitis_Attack | Previous cholecystitis attack: 0/1 |
| Previous_ERCP | Previous endoscopic retrograde cholangiopancreatography: 0/1 |
| Previous_Upper_Abdominal_Surgery | Previous upper abdominal surgery: 0/1 |
| WBC_10e9_L | Preoperative white blood cell count, ×10⁹/L |
| CRP_mg_L | Preoperative C-reactive protein, mg/L |
| Albumin_g_dL | Preoperative serum albumin, g/dL |
| NLR | Neutrophil-to-lymphocyte ratio |
| Gallbladder_Wall_mm | Ultrasonographic gallbladder wall thickness, mm |
| Pericholecystic_Fluid | Ultrasonographic pericholecystic fluid: 0/1 |
| Impacted_Stone | Impacted stone in the gallbladder neck/Hartmann's pouch: 0/1 |
| Max_Stone_Size_mm | Maximum stone diameter, mm |
| CBD_Diameter_mm | Common bile duct diameter, mm |
| Operation_Time_min | Total operative time, minutes |
| Subtotal_Cholecystectomy | 0/1 |
| Conversion_to_Open | Conversion to open surgery: 0/1 |
| Fundus_First | Fundus-first dissection: 0/1 |
| Extra_Port | Additional port placement: 0/1 |
| Difficult_LC | Primary outcome (composite, see Section 2.3): 0/1 |
| Drain_Placement | 0/1 |
| Gallbladder_Perforation | 0/1 |
| Postop_Bile_Leak | 0/1 |
| Intraabdominal_Collection | 0/1 |
| Postop_ERCP | 0/1 |
| Reoperation | 0/1 |
| Length_of_Stay_days | Length of hospital stay, days |
| Readmission_30d | 30-day readmission: 0/1 |
| Major_Complication | Clavien-Dindo grade ≥III complication: 0/1 |
| Mortality_30d | 30-day mortality: 0/1 |
| CAR | CRP-to-albumin ratio |

# **Supplementary Table S2. Preliminary, illustrative point-based risk score**

To translate the multivariable model into a form that could plausibly support bedside risk estimation, we derived a simple integer point score from the five independent predictors of the primary multivariable model (impacted stone, gallbladder wall thickness, previous cholecystitis attack, CRP, and BMI), with points assigned proportionally to each variable's regression coefficient, anchored to previous cholecystitis attack (2 points). In this cohort, the score discriminated difficult LC with an apparent (in-sample) AUC of 0.777, and observed difficult LC rates rose progressively across score quartiles (4.3%, 9.9%, 22.2%, and 40.7% from lowest to highest quartile). This score is exploratory, was not independently validated, and is presented solely to illustrate the potential for a multidomain preoperative score; it requires external, prospective validation before any clinical application and should not be used to guide clinical decisions in its current form.

| **Predictor** | **Points** |
| --- | --- |
| Impacted stone present | +3 |
| Previous cholecystitis attack | +2 |
| Gallbladder wall thickness, per mm | +1 |
| BMI, per 5 kg/m² | +1 |
| CRP, per 10 mg/L | +1 |

*Points sum to a total score; apparent (in-sample) AUC 0.777 in this cohort. Exploratory and not externally validated.*

# **Supplementary Table S3. TRIPOD Reporting Checklist**

This manuscript was prepared, where applicable to a single-center development study without an external validation cohort, in accordance with the relevant recommendations of the TRIPOD (Transparent Reporting of a multivariable prediction model for Individual Prognosis Or Diagnosis) statement. The table below maps core TRIPOD items to their location in the manuscript.

| **Section** | **Item** | **Reported on** |
| --- | --- | --- |
| Title | Identify as a prediction model study; target population; outcome | Title page; Title |
| Abstract | Structured summary of objectives, design, setting, participants, predictors, outcome, statistical analysis, results, conclusions | Abstract |
| Background/objectives | Rationale, including prior models; study objectives, including hypothesis | Introduction, final paragraph |
| Source of data | Study design and dates of recruitment | Section 2.1 |
| Participants | Eligibility criteria for participants | Section 2.2 |
| Outcome | Definition and method of outcome ascertainment | Section 2.3 |
| Predictors | Definition and method of measurement of predictors | Section 2.4; Supplementary Table S1 |
| Sample size | Explanation of how sample size was arrived at | Section 2.2 / Section 3.1 |
| Statistical analysis methods | Modeling method, handling of predictors, model-building procedures, internal validation method | Sections 2.5–2.6 |
| Risk groups/development-validation | Internal validation (resampling), performance measures (discrimination, calibration) | Sections 2.5–2.6 |
| Participants (results) | Flow of participants, characteristics, comparison of groups | Section 3.1; Table 1 |
| Model development | Unadjusted and adjusted associations of predictors with outcome | Sections 3.2–3.3; Tables 1–2; Figure 1 |
| Model specification | Full model or how to obtain predictions for individuals | Sections 2.5, 3.3; Supplementary Table S2 |
| Model performance | Discrimination and calibration measures, with confidence intervals | Sections 3.4, 3.7; Tables 3, 5; Figures 2–3 |
| Limitations | Study limitations, including sources of potential bias, generalizability, external validation | Section 4.1 |
| Interpretation | Overall interpretation, considering intended use and clinical usefulness | Discussion; Conclusion |
| Supplementary information | Availability of supplementary resources | Declarations; Supplementary Material |

# **Supplementary Table S4. STROCSS 2021 Reporting Checklist**

This manuscript was prepared in accordance with the STROCSS 2021 guideline for cohort studies in surgery. The table below maps each core reporting item to its location in the manuscript.

| **Item** | **Topic** | **Description** | **Reported on** |
| --- | --- | --- | --- |
| 1 | Title | Title identifies the study design and topic | Title page |
| 2 | Abstract | Structured summary of design, methods, results, conclusions | Abstract |
| 3 | Introduction — background/rationale | Scientific background and rationale | Section 1 |
| 4 | Introduction — objectives | Specific objectives/hypotheses | Section 1 (final paragraph) |
| 5 | Methods — study design | Key elements of study design presented early | Section 2.1 |
| 6 | Methods — setting | Setting, locations, relevant dates | Section 2.1–2.2 |
| 7 | Methods — participants | Eligibility criteria, sources, methods of selection | Section 2.2 |
| 8 | Methods — variables | Outcomes, exposures, predictors, confounders defined | Sections 2.3–2.4; Supplementary Table S1 |
| 9 | Methods — data sources/measurement | Sources of data and details of measurement | Section 2.4 |
| 10 | Methods — bias | Efforts to address potential sources of bias | Sections 2.2, 4.1 |
| 11 | Methods — study size | How study size was arrived at | Section 2.2 |
| 12 | Methods — statistical methods | All statistical methods, including subgroup/sensitivity analyses | Section 2.5 |
| 13 | Results — participants | Numbers at each stage of study | Section 3.1 |
| 14 | Results — descriptive data | Characteristics of participants and exposures | Section 3.2, Table 1 |
| 15 | Results — outcome data | Numbers of outcome events or summary measures | Sections 3.3–3.5, Tables 2–4 |
| 16 | Results — main results | Unadjusted and adjusted estimates and precision | Sections 3.2–3.4, Tables 1–3 |
| 17 | Discussion — key results, limitations, interpretation, generalizability | Summary of key results with reference to study objectives; limitations; cautious overall interpretation; generalizability | Sections 4–5 |

# **Supplementary Table S5. Bootstrap methodology and detailed confidence interval results**

*Added in response to Reviewer 1 (comment R1.5) and Reviewer 2 (comment R2.1), Round 1 revision.*

## **S5.1 Methodology**

All 95% confidence intervals (CIs) reported in Table 3, Table 5, and Table 6 of the main manuscript were derived using a patient-level bootstrap procedure with 1,000 resamples (sampling with replacement, resample size = original cohort size). For each resample, the complete stratified 5-fold cross-validation pipeline described in Methods Section 2.5 — including within-fold predictor standardization, logistic regression model fitting, and pooling of out-of-fold predicted probabilities — was re-executed in full on the resampled data. This full-pipeline re-execution (rather than resampling of the out-of-fold predictions alone) ensures that the reported uncertainty reflects both sampling variability in the underlying cohort and variability introduced by the cross-validation fold assignment itself.

For the paired ΔAUC confidence intervals, the three nested models (clinical, clinical+laboratory, combined+USG) were evaluated on the identical bootstrap resample in each of the 1,000 iterations, and the pairwise differences were computed within each resample before taking percentiles. This paired design accounts for the correlation between nested models trained on the same patients and yields a narrower, more appropriate interval than treating the two AUC estimates as independent.

Bootstrap resamples in which the resampled outcome vector contained fewer than the pre-specified minimum number of events in either class (8 events for the primary and non-temporal sensitivity analyses; 5 events for the smaller acute-cholecystitis subgroups) were excluded from the percentile calculation to avoid degenerate model fits; this affected a small minority of resamples and did not materially change the resulting intervals.

Random seed: NumPy RandomState seed 2026 was used for the outer bootstrap resampling loop; a fold-assignment seed of 42 (offset by the bootstrap iteration index) was used for the inner stratified 5-fold splits, to avoid identical fold assignments being reused across all bootstrap iterations. Code is available from the corresponding author on reasonable request.

## **S5.2 Full bootstrap distribution summaries**

**Table S5a. Primary outcome (composite difficult LC, n=648, 125 events) — AUC bootstrap distribution (1,000 resamples).**

| **Model** | **Point estimate** | **Bootstrap mean** | **Bootstrap SD** | **2.5th pct** | **97.5th pct** |
| --- | --- | --- | --- | --- | --- |
| Clinical | 0.723 | 0.734 | 0.0255 | 0.687 | 0.786 |
| Clinical + Laboratory | 0.734 | 0.745 | 0.0250 | 0.700 | 0.796 |
| Combined + USG | 0.768 | 0.782 | 0.0230 | 0.742 | 0.831 |

*Bootstrap mean is modestly higher than the point estimate, a well-recognized small-sample bootstrap feature (“bootstrap optimism” in resampled data with replacement); this does not affect the percentile-based CI, which was used as the primary inferential summary rather than the bootstrap mean.*

**Table S5b. Paired-bootstrap ΔAUC distribution (1,000 resamples, same resample used across all three nested models).**

| **Comparison** | **Point ΔAUC** | **2.5th pct** | **97.5th pct** | **CI excludes zero?** |
| --- | --- | --- | --- | --- |
| Clinical → Clinical+Laboratory | +0.011 | −0.007 | +0.040 | No |
| Clinical+Laboratory → Combined+USG | +0.034 | +0.010 | +0.071 | Yes |
| Clinical → Combined+USG | +0.045 | +0.020 | +0.091 | Yes |

**Table S5c. Sensitivity analysis (non-temporal outcome definition, n=648, 52 events) — AUC bootstrap distribution (1,000 resamples).**

| **Model** | **Point estimate** | **2.5th pct** | **97.5th pct** |
| --- | --- | --- | --- |
| Clinical | 0.705 | 0.635 | 0.801 |
| Clinical + Laboratory | 0.716 | 0.641 | 0.808 |
| Combined + USG | 0.739 | 0.675 | 0.832 |

# **Supplementary Table S6. Exploratory subgroup analysis by acute cholecystitis status — full detail**

*Added in response to Reviewer 2 (comment R2.2), Round 1 revision.*

This subgroup analysis was performed in response to Reviewer 2's request to examine whether model performance was driven disproportionately by a particular operative-setting subgroup. Because information on elective versus emergency operative timing was not reliably recorded in the underlying institutional dataset, acute cholecystitis status at presentation (recorded reliably for all 648 patients) was used as the best available proxy for operative-setting heterogeneity. The Acute_Cholecystitis variable was excluded from each subgroup-specific model because it is, by construction, invariant (constant) within each subgroup and cannot contribute to within-subgroup discrimination.

**Table S6a. Subgroup characteristics and combined-model performance.**

| **Subgroup** | **n** | **Difficult LC** | **Event rate** | **Cross-validated AUC** | **95% CI (bootstrap, 1000 resamples)** |
| --- | --- | --- | --- | --- | --- |
| Acute cholecystitis present | 262 | 82 | 31.3% | 0.706 | 0.685–0.832 |
| Acute cholecystitis absent | 386 | 43 | 11.1% | 0.666 | 0.641–0.820 |
| Full cohort (reference) | 648 | 125 | 19.3% | 0.768 | 0.742–0.831 |

*Predictors retained in each subgroup model: age, sex, BMI, ASA class, diabetes, hypertension, previous cholecystitis attack, previous ERCP, previous upper abdominal surgery, WBC, CRP, albumin, NLR, gallbladder wall thickness, pericholecystic fluid, impacted stone, maximum stone size, CBD diameter (18 predictors; Acute_Cholecystitis excluded as noted above).*

Interpretation: point estimates in both subgroups were numerically lower than the full-cohort estimate, which is an expected consequence of restricting the analysis to a more homogeneous case-mix (reduced outcome variance within each subgroup reduces achievable discrimination even when the underlying model is unchanged). The confidence intervals were wide and overlapping, and the exploratory analysis did not suggest that discrimination was confined to either subgroup. Given subgroup sample sizes (n=262 and n=386) and, in particular, the modest number of events in the non-acute subgroup (43 events across 5 cross-validation folds, averaging fewer than 9 events per fold), this analysis is underpowered to detect anything but a large difference in discrimination between settings, and should be treated as hypothesis-generating. We recommend that future prospective validation studies stratify enrollment or pre-specify a formal interaction test for acute-cholecystitis status (and, if available, elective/emergency operative setting) to definitively address this question.

Elective versus emergency operative setting: this information was not consistently or reliably coded in the retrospective institutional dataset underlying the present study (operative scheduling records did not reliably distinguish semi-elective from true emergency admissions across the full study period) and could therefore not be analyzed, either as a standalone stratification variable or as an additional covariate. This is noted as an explicit limitation in Section 4.1 of the main manuscript, and reliable prospective capture of this variable is recommended for future external validation work.
